# First record of *Clausidium* (Copepoda, Clausidiidae) from Brazil: a new species associated with ghost shrimps *Neocallichirus grandimana* (Gibbes, 1850) (Decapoda, Callianassidae)

**DOI:** 10.3897/zookeys.335.5490

**Published:** 2013-09-24

**Authors:** Terue C. Kihara, Carlos E. F. Rocha

**Affiliations:** 1 German Centre for Marine Biodiversity Research (DZMB), Senckenberg Research Institute, Südstrand 44, 26382 Wilhelmshaven, Germany; 2 Departamento de Zoologia, Instituto de Biociências, Universidade de São Paulo, Rua do Matão, trav. 14, no. 321, 05508-900, São Paulo, Brazil

**Keywords:** Biodiversity, CLSM, Crustacea, Poecilostomatoida, taxonomy, identification key

## Abstract

A new clausidiid copepod was found living in galleries of ghost shrimps *Neocallichirus grandimana* (Gibbes, 1850) in Natal, Brazil. The new species resembles to *Clausidium senegalense* Humes, 1957 and *Clausidium vancouverense* (Haddon, 1912) in the armature of P2–P5 of the female, and shares with *Clausidium senegalense* similar segmentation and armature of the antenna and maxilla of the female. Nevertheless, it can be easily distinguished from its congeners by the unique characteristics observed in the antenna, maxilliped and first leg of males, as well as by the anal somite, maxillule and maxilliped of the females. This new species extends the group distribution to the Southwest Atlantic and represents the first record of the genus in Brazil. A key for the identification of the species based on females of *Clausidium* is provided.

## Introduction

Clausidiids characterized by the presence of sucking discs on endopods of legs 1 to 4, the genus *Clausidium* Kossmann, 1874 was established to accommodate *Clausidium apodiforme* (Philippi, 1839). During the revision by Light and Hartman (1937), *Clausidium californiense* Wilson, 1935 was considered a synonym of *Clausidium vancouverense*, and at present this genus contains 10 species found in the Atlantic, Pacific and Indian oceans (Table 1). Wilson (1921) and Humes (1949) provided keys to species.

The representatives of *Clausidium* are typically external associates of marine decapods. They can be found inhabiting the burrows of ghost or mud shrimps of the families Callianassidae Dana, 1852 and Upogebiidae Borradaile, 1903 (Table 1).

**Table 1.** A list of species of *Clausidium* including known distributional records, hosts and references.

**Species**	**Distribution**	**Hosts**	**References**
*Clausidium apodiforme* (Phillippi, 1839)	North Atlantic Ocean	*Calianassa subterranea* (Montagu, 1808)	Philippi 1839
Syn: *Hersilia apodiformis* Phillipi, 1839	(Adriatic and	*Calianassa* sp.	Kossmann 1874
*Clausidium testudo* Kossman, 1874	Mediterranean sea)	*Pestarella candida* (Olivi, 1792)	Humes 1949
			Manning and Stevcic 1982
*Clausidium caudatum* (Say, 1818)	North Atlantic Ocean	*Callichirus major* (Say, 1818)	Say 1818
			Wilson C.B. 1921
			Pearse 1947
			Humes 1949
*Clausidium chelatum* Pillai, 1959	Indian Ocean	*Calianassa* sp.	Pillai 1959
*Clausidium dissimile* Wilson C.B., 1921	North Atlantic Ocean	*Calianassa* sp.	Wilson C.B. 1921
		*Gilvossius setimanus* (De Kay, 1844)	Wilson C.B. 1932
		*Lepidophthalmus louisianensis* (Schmitt, 1935)	Humes 1949
		*Sergio trilobata* (Biffar, 1970)	Corsetti and Strasser 2003
*Clausidium saldanhae* Kensley, 1974	South Atlantic Ocean	*Pestarella rotundicaudata* (Stebbing, 1902)	Kensley 1974
*Clausidium searsi* Wilson C. B.,1937	South Pacific Ocean	*Calianassa* sp.	Wilson C.B. 1937
			Humes 1949
*Clausidium senegalense* Humes, 1957	South Atlantic Ocean	*Calianassa* sp.	Humes 1957
*Clausidium tenax* Humes, 1949	North Atlantic Ocean	*Callichirus islagrande* (Schmitt, 1935)	Humes 1949
*Clausidium travancorense* Pillai, 1959	Indian Ocean	*Neocallichirus maxima* (A. Milne-Edwards, 1870)	Pillai 1959
*Clausidium vancouverense* (Haddon, 1912)	North Pacific Ocean	*Callichirus seilacheri* (Bott, 1955)	Haddon 1912
Syn: *Hersilia (Clausidium) vancouverensis*	South Pacific Ocean	*Neotrypaea californiensis* (Dana, 1854)	Humes 1949
Haddon, 1912		*Neotrypaea gigas* (Dana, 1852)	Light and Hartman 1937
*Clausidium californiense* Wilson C. B., 1935		*Upogebia pugettensis* (Dana, 1852)	Campos et al. 2009
			Iannacone et al. 2008
*Clausidium* sp.	South Pacific Ocean	*Callichirus seilacheri* (Bott, 1955)	Marin and George-Nascimento 1993, Hernáez and Wehrtmann 2007
***Clausidium rodriguesi*** sp. n.	South Atlantic Ocean	*Neocallichirus grandimana* (Gibbes, 1850)	

Although *Clausidium* is reported in population studies of the ghost shrimp *Callichirus seilacheri* (Bott, 1955) from Chilean coast (Marin and George-Nascimento 1993; Hernáez and Wehrtmann 2007), these species remain unidentified. *Clausidium searsi* Wilson, 1937 and *Clausidium vancouverense* (Haddon, 1912), collected along the Peruvian coast, are the only described species in South America.

A new clausidiid copepod, which can not be reconciled to any of the 10 species of *Clausidium* that have been described so far, was found living in galleries of ghost shrimps *Neocallichirus grandimana* (Gibbes, 1850) in the intertidal zone of a beach in Natal, state of Rio Grande do Norte (N.E. of Brazil). This is the first record of genus *Clausidium* in Brazil.

## Methods

The copepods were recovered from water drawn from the burrows and collected from pleopods of the ghost shrimp *Neocallichirus grandimana* in the intertidal zone of a beach in Natal, state of Rio Grande do Norte, Brazil (5°45'S, 35°11'W).

Whole specimens were examined in temporary lactic acid mounts. Chips of cover slip were used to support the cover glass of the preparation. After examination, material was returned to and preserved in 70% ethanol. Dissections were made in glycerine and the dissected parts were placed on slides and sealed with Glyceel.

A Leitz Laborlux D^®^ phase-contrast microscope and a Zeiss Axioskop 2 Plus^®^ compound microscope equipped with differential interference contrast, digital camera Nikon Coolpix 995^®^ and camera lucida were used to examine and prepare illustrations of the specimens.

Two females and two males were prepared for scanning electron microscopy (SEM). Specimens were dehydrated through a series of graded acetone; critical-point dried, mounted on stubs, sputter-coated with palladium and observed using a Philips XL 30 Field Emission Scanning Electron microscope (Philips, Eindhoven, Netherlands).

For confocal laser scanning microscopy (CLSM), a female was stained with Congo Red and mounted on slide following the procedure described by Michels and Büntzow (2010). The material was scanned using a Leica TCS SP5 (Leica, Wetzlar, Germany) equipped with a Leica DM5000 B upright microscope (Leica, Wetzlar, Germany) and 3 visible-light lasers (DPSS 10 mW 561 nm; HeNe 10 mW 633 nm; Ar 100 mW 458 nm, 476 nm, 488 nm and 514 nm), combined with the software LAS AF 2.2.1. - Leica Application Suite Advanced Fluorescence (Leica, Wetzlar, Germany).

Series of stacks were obtained, collecting overlapping optical sections throughout the whole preparation; the optimal number of scans and the imaging settings according to the software, are given in Table 2. Final images were obtained by maximum projection, and CLSM illustrations were composed and adjusted for contrast and brightness using the software Adobe Photoshop CS4 (Adobe Systems, San José, U.S.A.).

Total body length was measured from the anterior margin of the rostrum to the posterior margin of the caudal rami. The descriptive terminology follows Huys and Boxshall (1991) and Huys et al. (1996). Abbreviations used in the text are: ae, aesthetasc; P1–P6, legs 1–6; exp and enp, exopod and endopod respectively; exp (enp)-1 (-2, -3), proximal (middle, distal) segments of a ramus.

The type material is deposited in the collection of the Museu de Zoologia, Universidade de São Paulo, São Paulo, Brazil.

**Table 2.** Microscope lens and confocal laser scanning microscopy (CLSM) settings used for the observation of the specimens; Ch1 and Ch2 = detection channels 1 and 2.

**Lens**	**HC PL APO CS (High-grade colour-corrected Plan Apochromat lens for confocal)**
Objective	20×
Numerical aperture	0.7
Immersion	Oil
Excitation wavelength	488 and 633 nm
Laser intensity	50% and 33%, respectively
Excitation beam splitter	TD 488/561/633
Detected emission wavelength	Ch1: 493 – 600 nm
	Ch2: 650 – 750 nm
Detector gain	833.8 and 791.6 V
Amplitude offset	-0.9 and -1.0 %
Electronic zoom	3X
Pinhole aperture	54.6 μm
Image format	2048 × 2048 dpi

## Results

### Order Poecilostomatoida Burmeister, 1835
Family Clausidiidae Embleton, 1910
Genus *Clausidium* Kossmann, 1874

#### Clausidium rodriguesi sp. n.

http://zoobank.org/96A49C80-55DA-484C-AC7A-08A84A5AE21F

http://species-id.net/wiki/Clausidium_rodriguesi

Figs 1 – 11

##### Type material.

Holotype female (reg. no. MZUSP 16464) in ethanol, dissected paratypes consist of 2 females and 2 males (reg. no. MZUSP 19628–19631) undissected paratype consist of 1 female (reg. no. MZUSP 19632) deposited in the collection of the Museu de Zoologia, Universidade de São Paulo, São Paulo, Brazil. All material collected in 02/1984 from the type locality by Prof. Dr. G. Shimizu.

##### Type locality.

Rio Grande do Norte, Natal, margin of Potengi river (5°45'S, 35°11'W). All specimens from water drawn from the burrows and pleopods of the ghost shrimp *Neocallichirus grandimana*.

##### Description.

**Figures 1–3.**
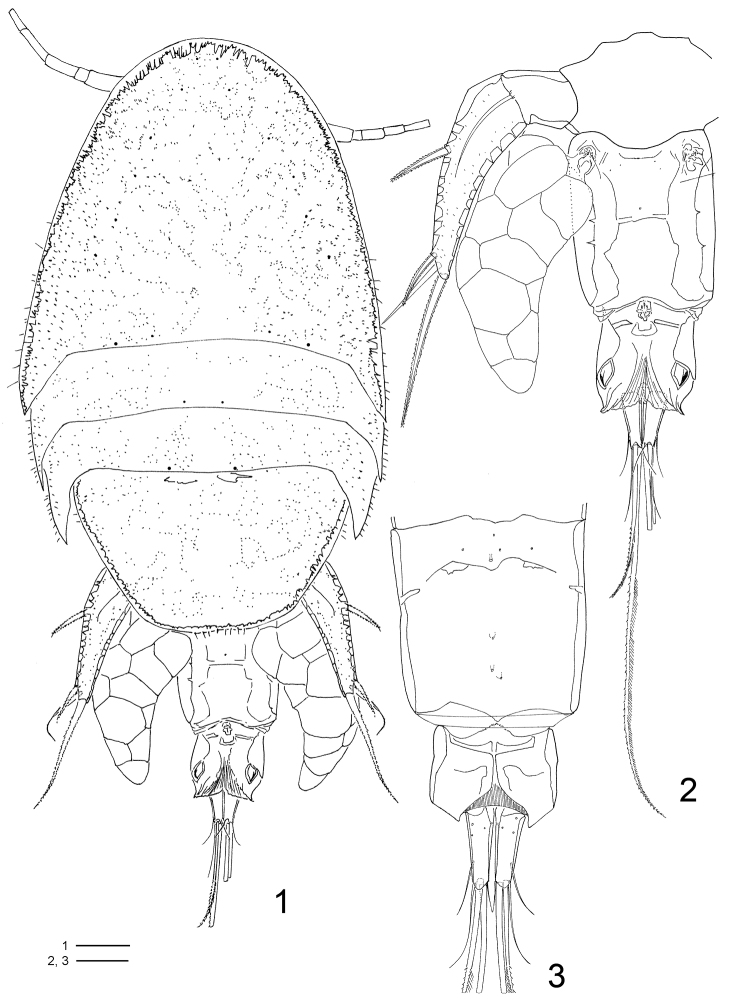
*Clausidium rodriguesi* sp. n. Female: **1** habitus, dorsal **2** urosome, dorsal **3** urosome lacking somite bearing P5, ventral. Scale bars: **1** = 100 μm; **2, 3** = 50 μm.

**Figures 4–6.**
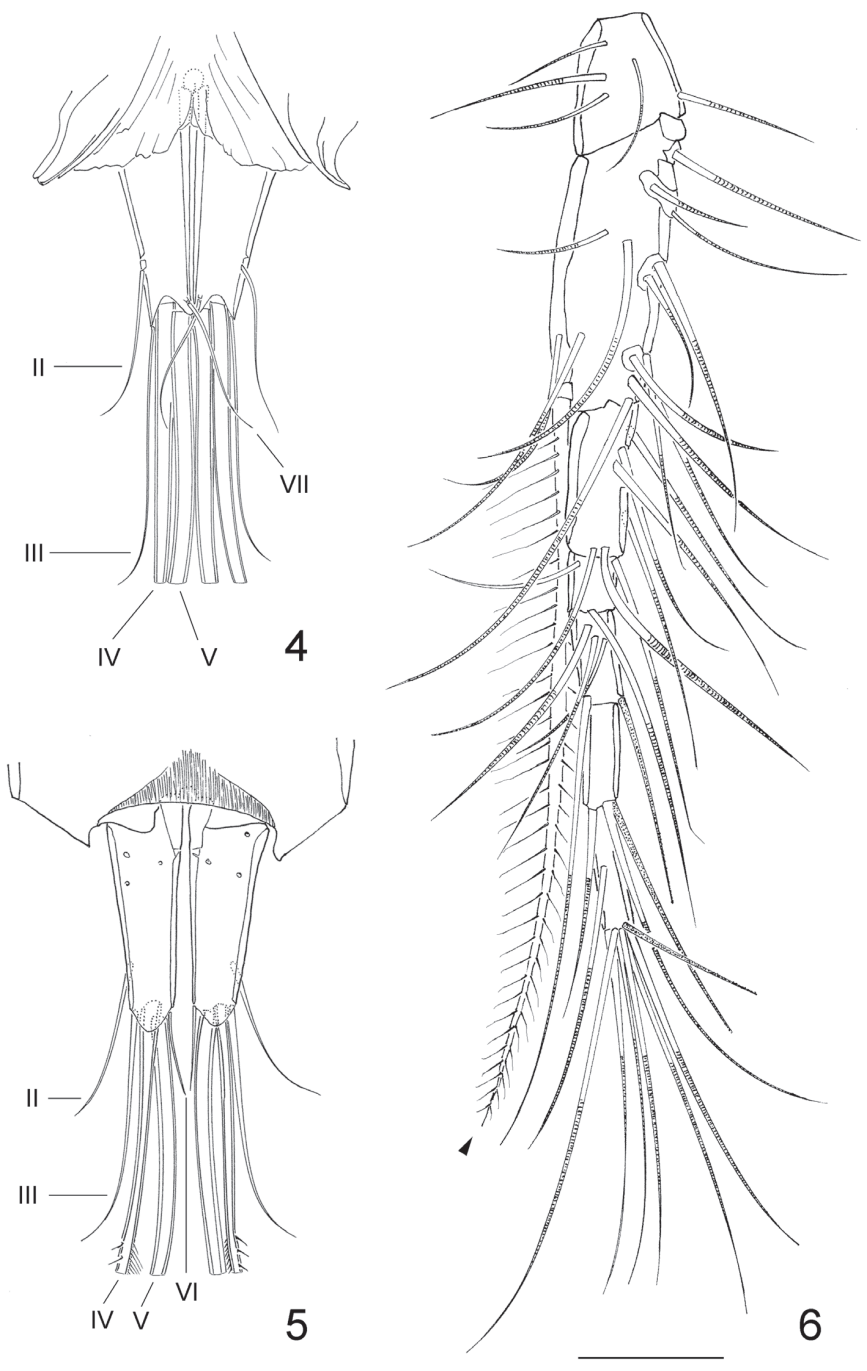
*Clausidium rodriguesi* sp. n. Female: **4** caudal rami, dorsal **5** caudal rami, ventral **6** antennule (arrow head indicating bipinnate seta). Scale bar = 50 μm.

**Figures 7–13.**
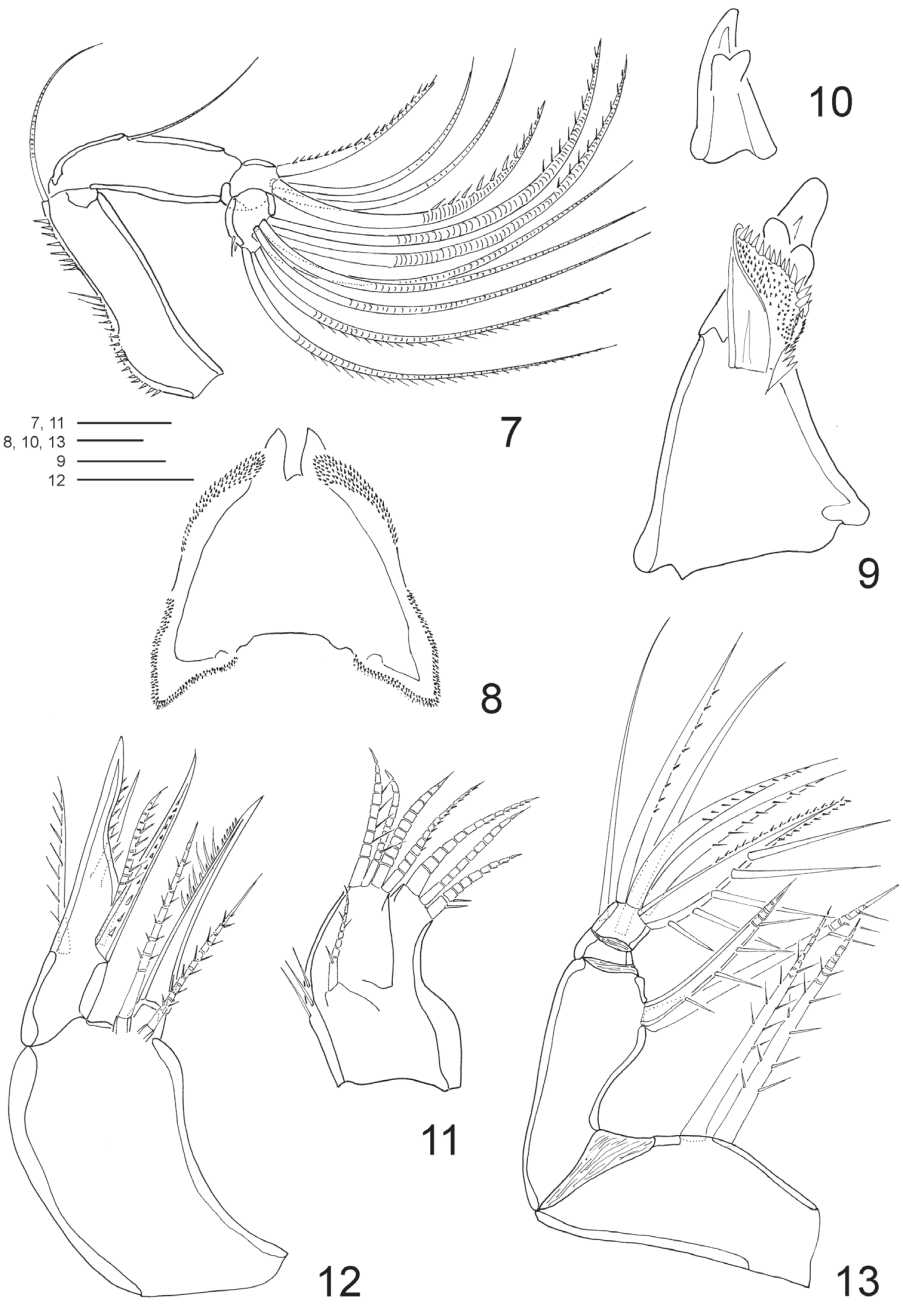
*Clausidium rodriguesi* sp. n. Female: **7** antenna **8** labrum **9** mandible **10** Detail of mandible tooth **11** maxillule **12** maxilla **13** maxilliped. Scale bars: **7** = 50 μm; **8** = 10 μm; **9, 10** = 25 μm; **11–13** = 20 μm.

**Figures 14–17.**
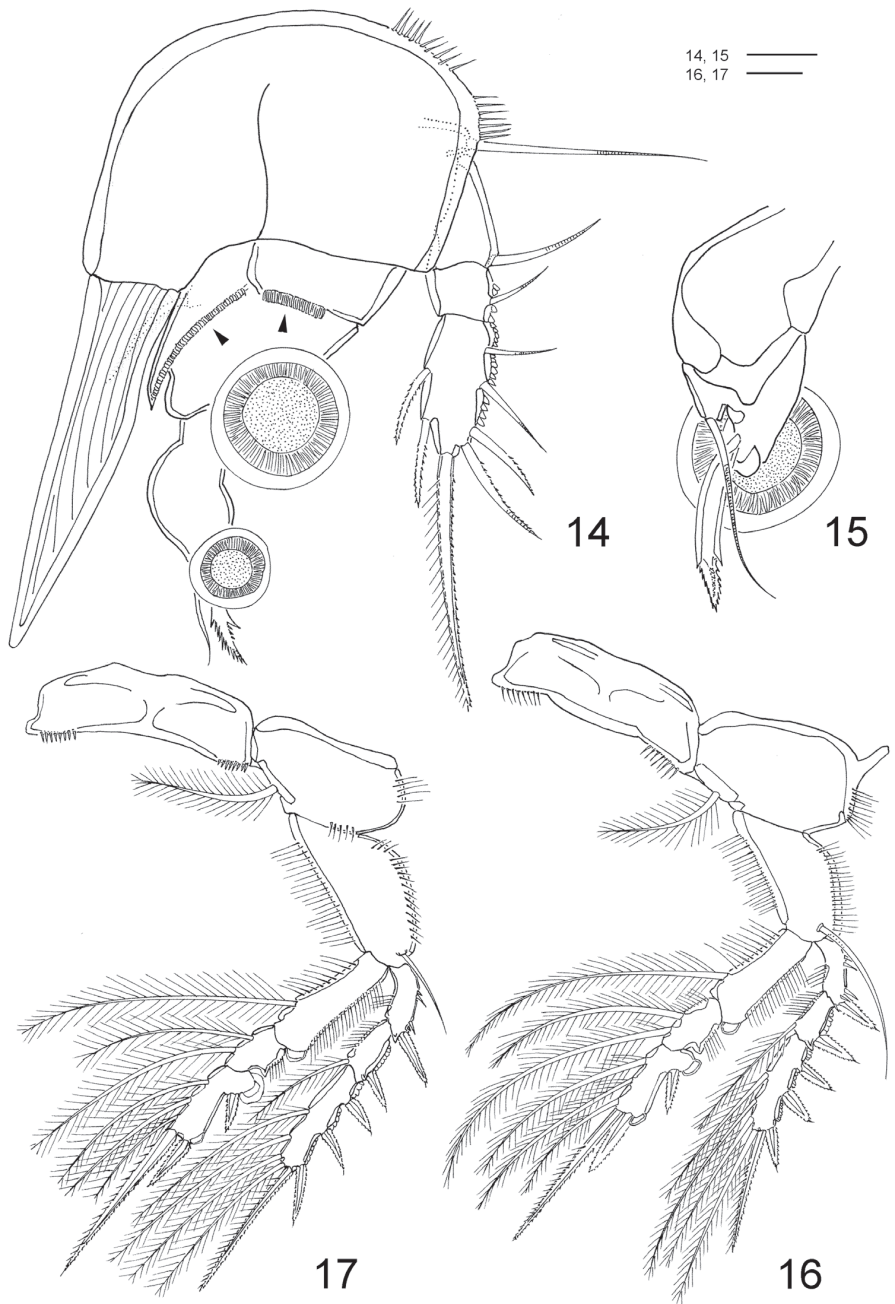
*Clausidium rodriguesi* sp. n. Female: **14** P1, anterior (arrows indicating adhesive fringe) **15** detail of distal area of P1 endopod, posterior **16** P2, anterior **17** P3, anterior. Scale bars: **14** = 20 μm; **15** = 10 μm; **16, 17** = 50 μm.

**Figures 18–20.**
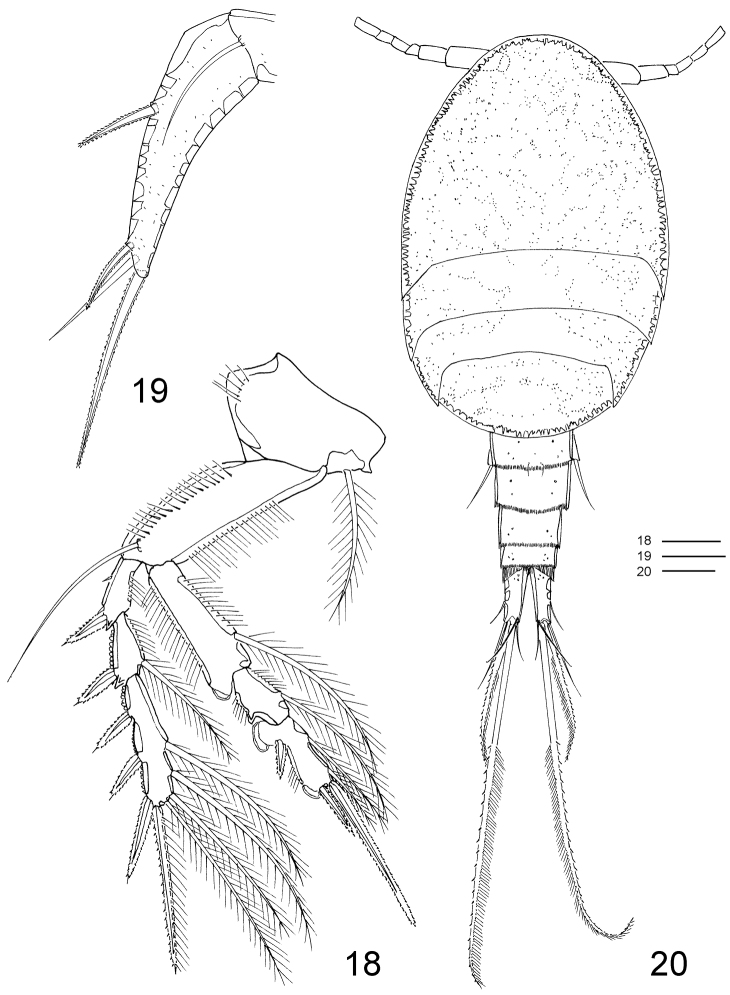
*Clausidium rodriguesi* sp. n. Female: **18** P4, anterior **19** P5, anterior. Male: **20** habitus, dorsal. Scale bars: **18** = 20 μm; **19** = 50 μm; **20** = 100 μm.

FEMALE (Figs 1–19, 32–38, 42, 45, 47, 48): Total length, excluding setae on caudal rami, 1.36–1.40mm (N=6). Body cyclopiform (Figs 1, 32–33), maximum width measured at posterior margin of cephalic shield. Prosome twice longer than urosome. First pedigerous somite fused with cephalosome. Body prosomites with minute integumental pits, sensilla and numerous pores distributed as illustrated in Fig. 1. Somites bearing P2–P3 subequal, both with latero-posterior margin sharply drawn out and posterior margin smooth. Somite bearing P4 trapezoid in form, longer than the two anterior somites combined, posterior margin with row of sensilla. Urosome (Figs 1–3, 32–35) 3-segmented, distinctly narrower than prosome. Urosome comprising fifth pedigerous somite, genital double-somite, and anal somite. Somite bearing P5 (Figs 1–2, 32–33) 1.4 times broader than long in dorsal view and with P5 arising ventrolaterally. Genital double-somite (Figs 1–3, 32–33) 1.3 times longer than broad, dorsal and ventro-lateral cuticular ridges marking plane of fusion between genital and first abdominal somite. Genital apertures (Fig. 2) located dorsolaterally on each side, near posterior margin of fifth pedigerous somite. Presence of pairs of pores near genital apertures and medial pore on dorsal view. Ventral surface with pores along medial region (Fig. 3). Egg sacs dorsolaterally located on each side, reaching posterior edge of anal somite and containing 13–15 eggs each. Anal somite (Figs 1–3, 32–33) well developed, formed by second to fourth abdominal somites fused in single somite; dorsal surface with well sclerotized leaf-like areas laterally displaced and intricate folders as illustrated in Figures 2 and 34, clearly incised medially, posterior borders with pointed curved extensions on outer corners; almost quadrate in ventral view, with pointed posterior inner corners and fringed with membrane medially.

Caudal ramus (Figs 2–5) about 3.5 times longer than wide, and armed with 6 setae. Seta I absent, setae II and III slender and naked; setae IV and V strongly developed and bipinnate, plumose on inner edge and spinulose on outer edge (seta V, 2.5 times longer than seta IV); seta VI the shortest; seta VII triarticulate and located at inner posterior corner, both naked. Caudal ramus with rounded lappet on posterior margin of ventral surface covering basal portion of setae III–V.

Rostrum (Fig. 33) incorporated into cephalothorax, demarcated by sclerotized areas laterally; with pair of sensilla ventrally and pattern of pores as illustrated.

Antennule (Fig. 6) 7-segmented. Segment 2 longest, with well-developed pinnate seta inserted on inner distal corner and extending over tip of antennule (arrowed in Fig. 6). Aestethasc inconspicuous, very similar to other setae. Segment 6 with aesthetasc fused basally to seta. Armature formula: I-[5], II-[14 + 1 bipinnate], III-[6], IV-[3], V-[4 + ae], VI-[2 + ae], VII-[7 + ae].

Antenna (Figs 7, 36) 4-segmented. Coxobasis elongated, with row of spinules along inner margin, with single seta on inner distal corner. Endopod 3-segmented; segment 1 with seta along inner margin; segment 2 with 4 setae (2 pinnate and 2 naked); segment 3 with row of spinules along distal margin, 7 apical setae, 2 of them with setules and 2 with spinules.

Labrum (Figs 8, 36, 39–40) twice wider than long; lateral projections with row of denticles. Metastomal area ornamented as in figures 40 and 41.

Mandible (Figs 9–10, 36) well developed. Armed with 3 elements, 1 toothed projection, 1 small seta, and 1 conical structure covered with minute spinules covering inner surface and spines along distal margin.

Maxillule (Figs 11, 36) bilobed, with 1 lateral seta pinnate. Outer lobe with row of spinules along outer margin and 4 setae (2 pinnate and 2 naked). Inner lobe with 3 setae (2 pinnate and 1 naked).

Maxilla (Figs 12, 36) 2-segmented. Syncoxa with 2 bipinnate setae and one stout spine with spinules on distal edge. Basis with large spinous process with spinules along concave margin, bearing 3 setae (2 pinnate and 1 naked) and 1 pinnate spine.

Maxilliped (Figs 13, 36) 4-segmented. Syncoxa with 2 bipinnate setae along inner margin. Basis with 1 pinnate seta and 1 spine with long spinules. Endopod 2-segmented; first segment unarmed; second segment bearing 2 naked lateral setae, 3 pinnate distal setae and stout distal spine with long and slender spinules along inner margin; minute spinules on opposite margin.

P1 (Figs 14–15, 33, 37, 42) biramous, both rami 3-segmented, and highly modified for prehension. Coxa and basis fused forming well-developed segment with row of spines along proximal margin, plumose seta on outer proximal corner; large blade-like seta with acute apex and concentric lines on inner distal corner. Exp-1 and -2 with 1 outer seta each; exp-2 with row of denticles along outer margin. Exp-3 with row of denticles along outer margin, 3 outer setae (2 naked and 1 pinnate), 2 apical setae (outer one pinnate, inner one longest and bipinnate) and 2 inner bipinnate setae. Enp-1 with 1 stout curved process with an adhesive fringe (arrowed in Fig. 14). Enp-2 with pinnate seta. Enp-3 elongated, irregular segment ending in a lobe with serrate margin and armed with 1 seta and 2 sucking discs (Fig. 15); proximal sucking disc 1.6 larger than distal one.

P2–P4 (Figs 16–18, 33, 38, 47) biramous, with both rami 3-segmented. Coxae with inner plumose seta, row of sparse setules along outer margin and row of spinules along distal margin (P3). Basis of P2–4 longitudinally elongate, with naked seta on outer distal corner, row of spinules along outer margin and row of setules along inner margin. Exp-1 and Exp-2 with row of setules along inner margin and row of spinules (exp-1) or denticles (exp-2) along outer margin, but exp-3 with denticles along outer margin; exopod outer spines serrate and with terminal flagellum (Fig. 48), apical spine with serrate outer margin and spinulose inner margin. Enp-1 and Enp-2 with row of setules along both margins; endopod outer andapical spines serrate and with terminal flagellum, inner apical spines with serrate outer margin and spinulose inner margin (P2–P3) (Figs 16, 17) or both margins serrate (P4) (Fig. 18); sucking discs (Fig. 47, detail in Fig. 49) on distal inner edge of enp-1 and proximal and subterminal inner edges of enp-3.

Armature formula of P2–P4 (Figs 13–16) as follows (Roman numerals representing spines, Arabic numerals representing setae):

P5 (Figs 2, 19, 33) uniramous, 2-segmented and located laterally on somite. Protopod with 1 outer seta; free exopodal segment elongated with 2 serrate spines, 1 naked seta along outer margin and serrate spine apically; dorsal punctuations as in figures 19 and 33.

P6 (Fig. 2) consisting of 3 small setae.

**Figures 21–24.**
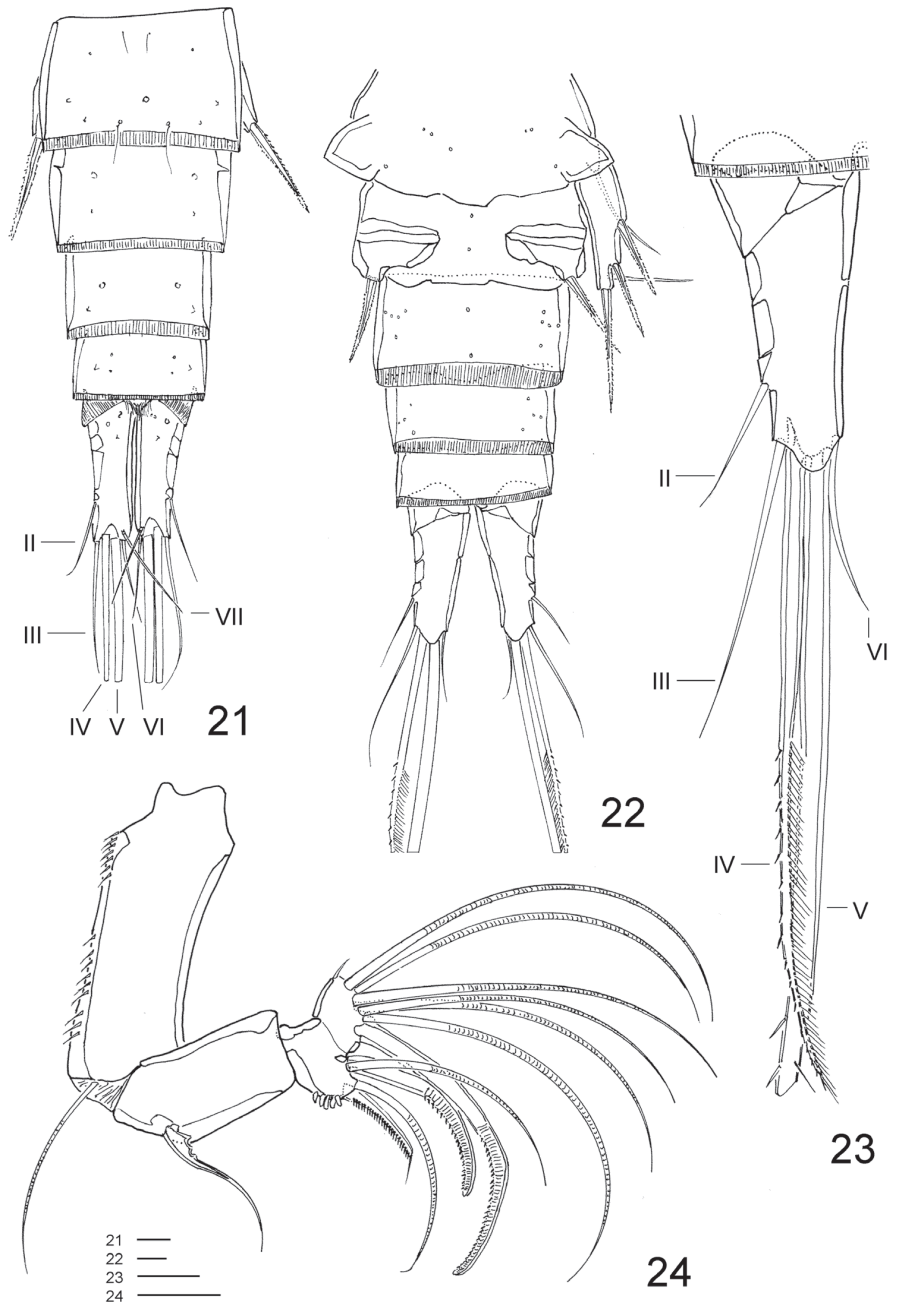
*Clausidium rodriguesi* sp. n. Male: **21** urosome lacking somite bearing P5, dorsal **22** urosome, ventral **23** caudal ramus, dorsal **24** antenna. Scale bars: **21–23** = 20 μm; **24** = 25 μm.

**Figures 25–29.**
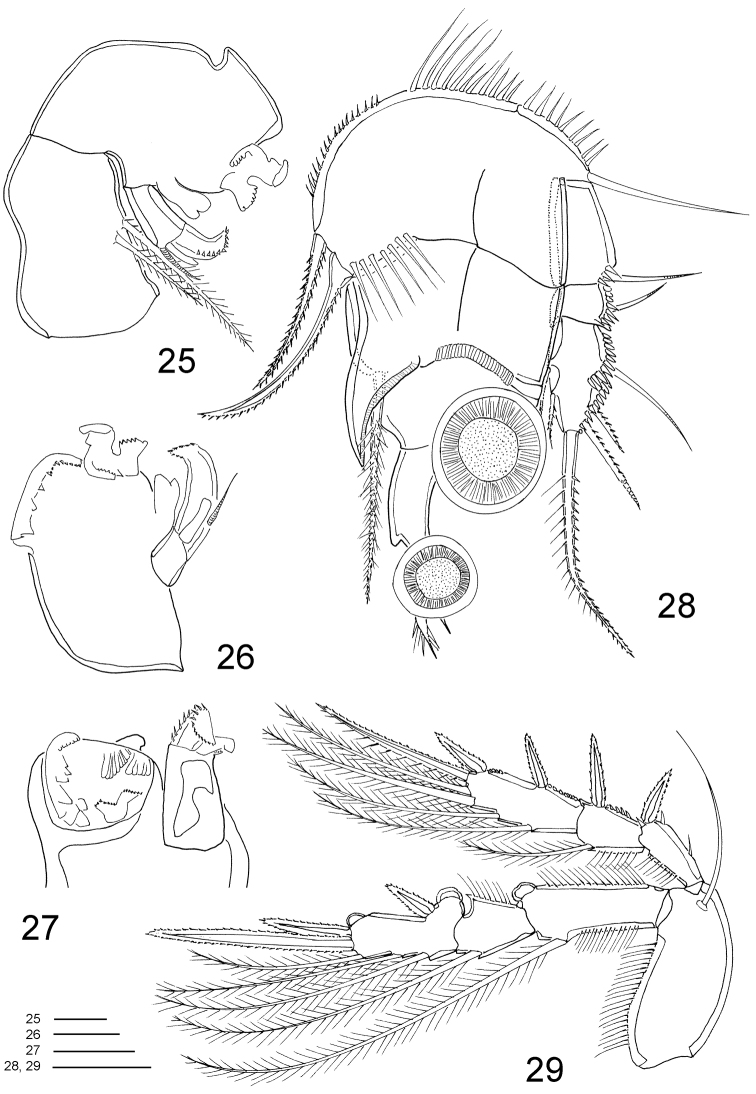
*Clausidium rodriguesi* sp. n. Male: **25** mandible **26** mandible, detail, ventral **27** mandible, detail, dorsal **28** P1, anterior **29** P2, anterior. Scale bars: **25–27** = 10 μm; **28, 29** = 20 μm.

**Figures 30–31.**
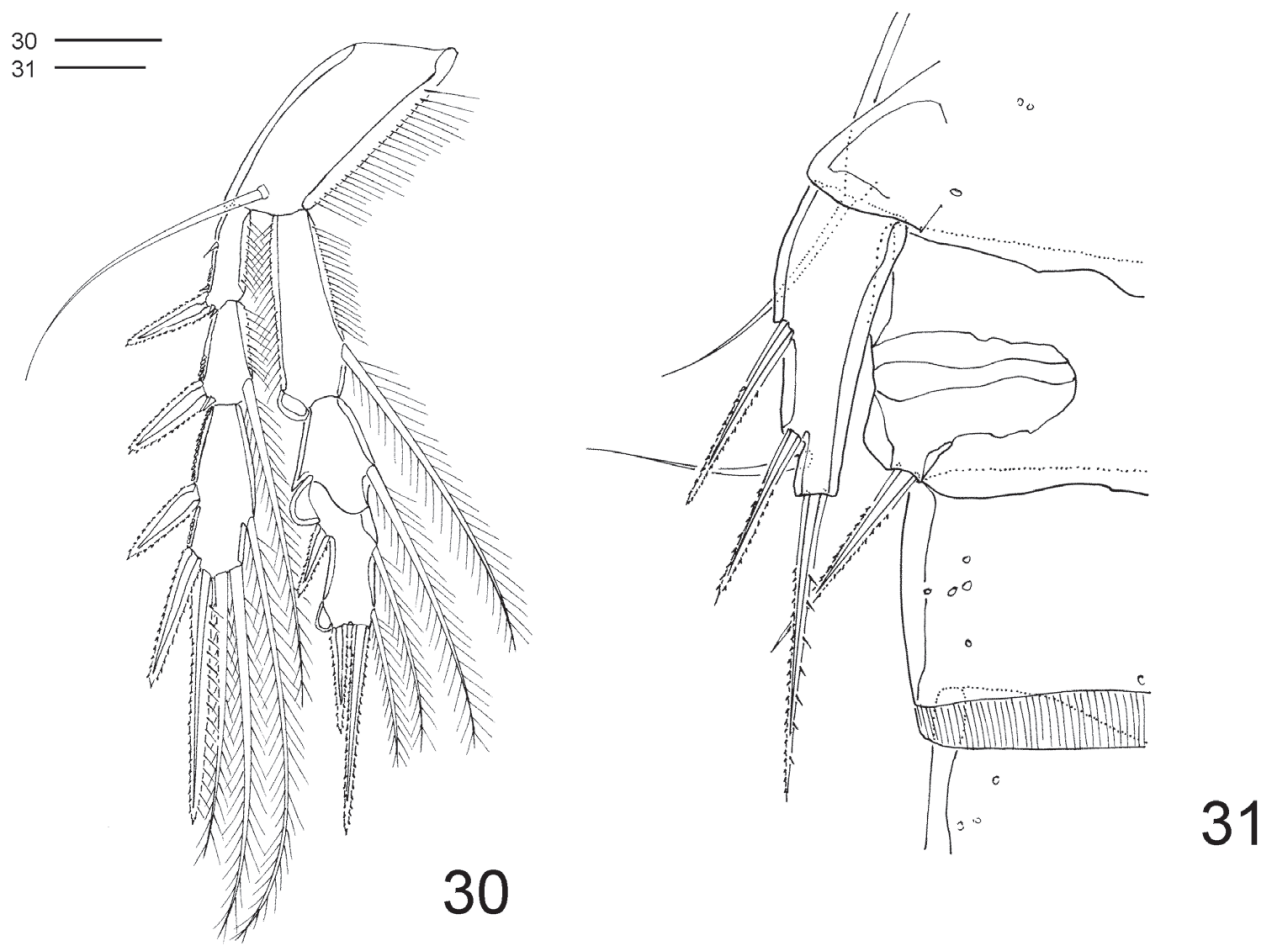
*Clausidium rodriguesi* sp. n. Male: **30** P4, anterior **31** P5 and P6. Scale bar: 20 μm.

MALE (Figs 20–31, 39–41, 43, 44, 46): Total length, excluding setae on caudal rami, 0.75–0.79mm (N=4). Body cyclopiform (Fig. 20). Prosome longer than urosome (1.5:1). First pedigerous somite fused with cephalosome. Body prosomites with minute integumental pits, sensilla and numerous pores distributed as illustrated in Figure 20. Cephalosome and 3 free prosomites with posterior borders smooth; somites bearing P2–P3 subequal; somite bearing P4 with distal margin rounder than in female. Urosome (Figs 21–22) 6-segmented, distinctly narrower than prosome. Somite bearing P5 (Fig. 22) 1.5 times broader than long in ventral view and with P5 arising ventrolaterally. Pores and sensilla as illustrated in Figures 21–22. Hyaline frills of first to third abdominal somites finely striated. Anal somite (Figs 21–22) extremely reduced and deeply incised medially, with hyaline frill on dorsal posterior margin.

**Figures 32–33.**
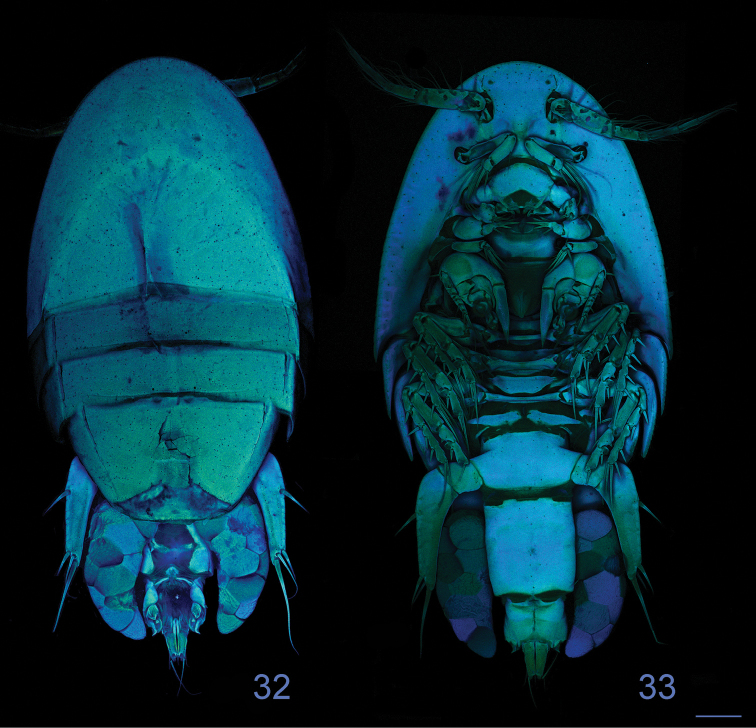
*Clausidium rodriguesi* sp. n. Female: Confocal laser scanning microscopy maximum projections**33** habitus, dorsal **34** habitus, ventral. Scale bars: 100 μm.

**Figures 34–38.**
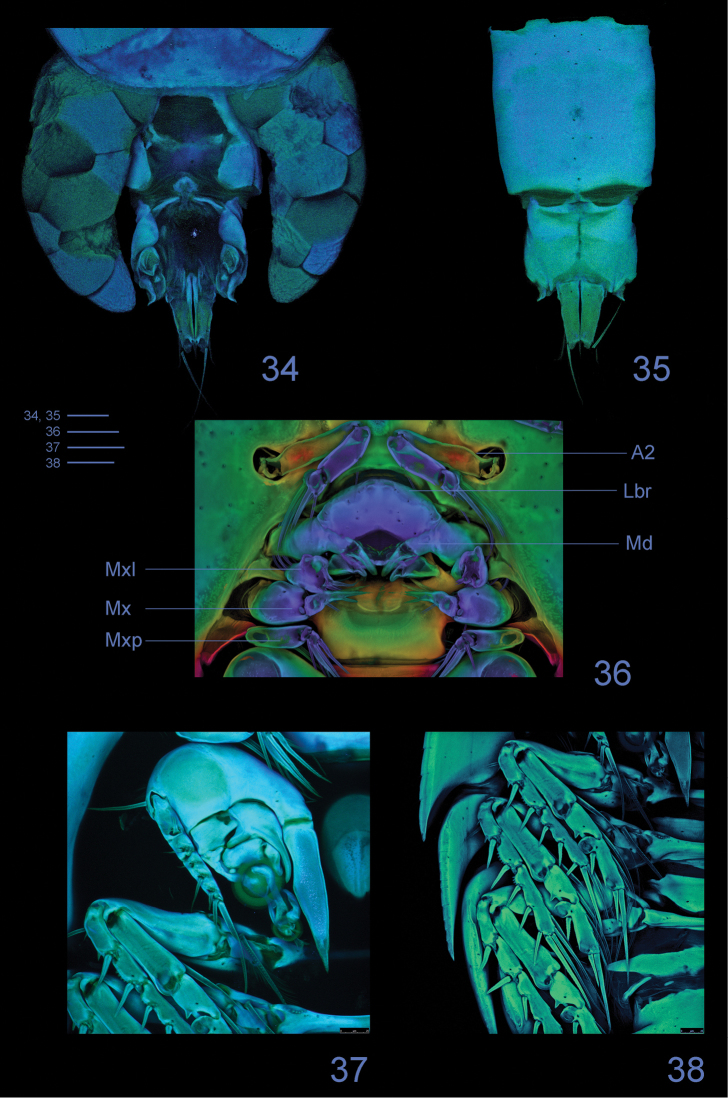
*Clausidium rodriguesi* sp. n. Female: Confocal laser scanning microscopy maximum projections **34** urosome, dorsal **35** urosome lacking somite bearing P5, ventral **36** antenna and oral region **37** P1, anterior **38** P2-P4, anterior. Scale bars: 50 μm.

**Figures 39–49.**
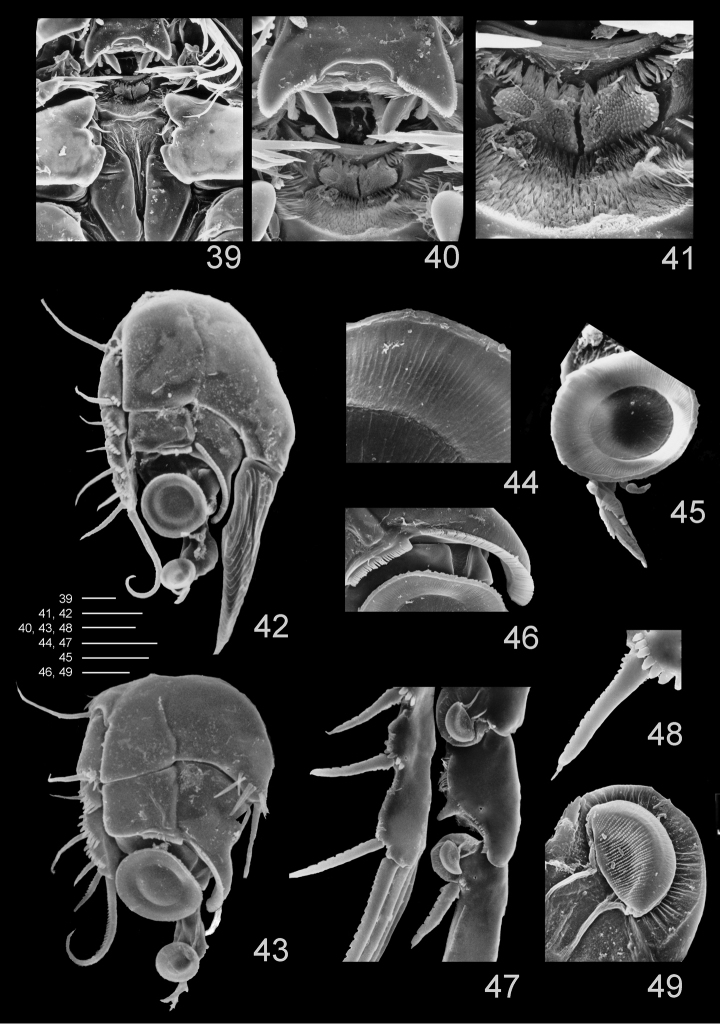
*Clausidium rodriguesi* sp. n.: Scanning electron microscopy photos **39** metastomal area, male **40** detail of metastomal area, male **41** detail of metastomal area, male **42** P1, anterior, female **43** P1, anterior, male **44** detail of sucking disc of P1, male **45** detail of lobe with serrate margin and distal sucking disc of enp-3 of P1, female **46** detail of P1 enp-1 adhesive fringe, male **47** sucking discs of P2, female **48** detail of serrate spine with apical flagellum of P2, female **49** detail of sucking disc of P2, female. Scale bars: **39, 40, 47** = 25 μm; **41, 44–46** = 10 μm; **42** = 35 μm; **43** = 20 μm; **48** = 12.5 μm; **49** = 4 μm.

Caudal ramus (Figs 21–23), antennule, mandible, maxillule and maxilla resembling those of female.

Antenna (Fig. 24) 4-segmented. Coxobasis elongated, with row of spinules along inner margin, with single seta on inner distal corner. Endopod 3-segmented; seta on segment 1 with proximal third enlarged and irregular, inserted along inner margin; segment 2 with row of denticles, 2 naked setae, spinulose spine with terminal flagellum and curved spine with serration along distal inner margin; segment 3 with row of spinules along distal margin, 6 naked setae and 1 curved spine with serration along distal inner margin.

Maxilliped (Figs 25–27, 39) well developed, strongly modified. Syncoxa with 2 pinnate setae. Basis with unequal denticulate projections and distal half of border curved and with irregular margin. Endopod 1-segmented; with strong serrate claw implanted near curved projection, and 1 small seta.

P1 (Figs 28, 43) similar to female. Coxa and basis fused, with rows of stout spinules along proximal margin, row of long spinules near inner distal corner, long naked seta on outer edge and 2 pinnate setae on inner distal corner. Exp-1 and Exp-2 with 1 outer seta each, and row of spinules along outer margin. Exp-3 with row of denticles along outer margin, 3 outer setae (2 pinnate and 1 naked), 1 apical bipinnate seta and 2 inner bipinnate setae. Enp-1 with adhesive fringe along distal margin, stout curved process with adhesive fringe (Fig. 46) and long pinnate seta on inner distal corner. Enp-2 and Enp-3 as in female.

P2–P4 (Figs 29–30) lacking outer spine on Exp-3. P4 (Fig. 30) without inner seta on Exp-3. Armature formula of P2–P4 as follows (Roman numerals representing spines, Arabic numerals representing setae):

P5 (Fig. 31) smaller than in female.

P6 (Fig. 31) represented by membranous flaps with bipinnate seta.

##### Variability.

One female paratype showed left P3 endopod modified - enp-2 with only 1 seta and enp-3 with 8 elements in total (I,II, I+4).

##### Etymology.

The new species is named in honor of Prof. Dr. Sérgio de A. Rodrigues (Universidade de São Paulo) in recognition of his significant contributions to the taxonomy of Callianassidae and who kindly made available the studied material.

## Discussion

Although the new species *Clausidium rodriguesi* resembles *Clausidium senegalense* and *Clausidium vancouverense* in the armature of P2–P5 of the female, and shares with *Clausidium senegalense* similar segmentation and armature of the female antenna and maxilla, it can be easily distinguished from its congeners by the unique characteristics observed in the males, - i. e., antenna with modified elements (enlarged seta on endopod-1 and spines on endopod-2 and endopod-3); maxilliped with distinct denticulate projections; and P1 coxobasis with 1 outer and 2 inner setae.

Other differential features refer to the morphology of the anal somite with sclerotized leaf-like areas and intricated folders dorso-laterally, posterior borders with pointed curved extensions on outer corners and clearly incised medially; maxillule with apical outer lobe bearing 2 pinnate and 2 naked setae, as well as maxillular inner lobe bearing 2 pinnate and 1 naked setae; and female maxilliped with 1 pinnate seta and 1 pinnate spine on basis, and endopod-2 bearing 2 naked lateral setae and 4 pinnate apical elements (3 setae and 1 spine).

This new species, the first record of *Clausidium* in Brazil, not only extends the group distribution to the Southwest Atlantic but also enlarges the host list for the genus by adding *Neocallichirus grandimana* (Gibbes, 1850).

A dichotomous key to the 11 valid species of *Clausidium* based exclusively on females is given below. Males differ from females by shape and size of the body, as well as by small differences in the segmentation and armature of antenna, setal formulae of legs, maxilliped strongly modified - adapted to grasp the female. In addition, many records of males are not well detailed. Thus, any identification of males must be verified against the best available description of the species.

**Table:** 

1	Antenna 3-segmented	2
–	Antenna 4-segmented	3
2	Antennary distal segment with 5 setae	*Clausidium caudatum* (Say, 1818)
–	Antennary distal segment with 7 setae	*Clausidium searsi* C. B. Wilson, 1937
3	P2 and P3 enp-2 with 1 seta	4
–	P2 and P3 enp-2 with 2 setae	5
4	P1 exp-3 with 5 elements in total	*Clausidium saldanhae* Kensley, 1974
–	P1 exp-3 with 7 elements in total	*Clausidium tenax* Humes, 1949
5	P4 enp-2 with 1 seta	6
–	P4 enp-2 with 2 setae	7
6	Maxilliped enp-2 with 5 elements in total; antenna enp-2 with 3 setae	*Clausidium vancouverense* (Haddon, 1912)
–	Maxilliped enp-2 with 6 elements in total; antenna enp-2 with 4 setae	*Clausidium rodriguesi* sp. n.
7	P2 and P3 enp-3 with 5 elements in total	*Clausidium apodiforme* (Phillippi, 1839)
–	P2 and P3 enp-3 with 6 elements in total	8
8	P4 exp-3 with 9 elements in total	*Clausidium travancorense* Pillai, 1959
–	P4 exp-3 with 8 elements in total	9
9	P1 exp-3 with 4 elements in total	*Clausidium dissimile* C. B. Wilson, 1921
–	P1 exp-3 with 5 elements in total	*Clausidium chelatum* Pillai, 1959
–	P1 exp-3 with 7 elements in total	*Clausidium senegalense* Humes, 1957

## Supplementary Material

XML Treatment for Clausidium rodriguesi
